# A multimodal physiological and psychological dataset for human with mental stress induced myocardial ischemia

**DOI:** 10.1038/s41597-024-03462-2

**Published:** 2024-06-27

**Authors:** Xiaoting Peng, Dantong Li, Jun Quan, Chao Wu, Huixian Li, Entao Liu, Lianting Hu, Shuai Huang, Lingcong Kong, Xuanhui Chen, Huan Yang, Huiying Liang, Shuxia Wang, Huan Ma, Qingshan Geng

**Affiliations:** 1 Medical Big Data Center, Guangdong Provincial People’s Hospital (Guangdong Academy of Medical Sciences), Southern Medical University, Guangzhou, 510080 China; 2 Guangdong Provincial Cardiovascular Institute, Guangzhou, Guangdong Province 510080 China; 3 Guangdong Provincial Key Laboratory of Artificial Intelligence in Medical Image Analysis and Application, Guangdong Provincial People’s Hospital, Guangdong Academy of Medical Sciences, Guangdong Province, 510080 China; 4 Department of Nuclear Medicine, Guangdong Provincial People’s Hospital, Guangdong Academy of Medical Sciences, Guangzhou, China; 5 Department of Cardiology, Shenzhen People’s Hospital (The Second Clinical Medical College, Jinan University; The First Affiliated Hospital, Southern University of Science and Technology), Shenzhen, 518020 Guangdong China

**Keywords:** Cardiovascular diseases, Diagnosis

## Abstract

Accurate differentiation between angina with no obstructive coronary arteries (ANOCA) and mental stress-induced myocardial ischemia (MSIMI) is crucial for tailored treatment strategies, yet public data scarcity hampers understanding. Given the higher incidence of both conditions in women, this study prospectively enrolled 80 female ANOCA and 39 age-matched female controls, subjecting them to three types of mental stress tasks. ECGs were continuously monitored across Rest, Stress, and Recover stages of the mental stress tasks, with PET/CT imaging during the Stress stage to evaluate myocardial perfusion. With PET/CT serving as the gold standard for MSIMI diagnosis, 35 of the 80 ANOCA patients were diagnosed as MSIMI. Using ECG variables from different stages of mental stress tasks, we developed five machine learning models to diagnose MSIMI. The results showed that ECG data from different stages provide valuable information for MSIMI classification. Additionally, the dataset encompassed demographic details, physiological, and blood sample test results of the patients. We anticipate this new dataset will significantly push further progress in ANOCA and MSIMI research.

## Background & Summary

Angina with no obstructive coronary arteries (ANOCA) is increasingly recognized in contemporary populations especially in women, presenting a relatively higher risk for cardiac events, and posing significant management challenges^1,2^. Patients with ANOCA commonly endure a high burden of symptoms and may experience repeated presentations to multiple healthcare providers before receiving a diagnosis, which may exacerbate anxiety and depression^1^. Notably, recent research indicates that women with ANOCA are more susceptible to severe myocardial ischemia during periods of mental stress, known as mental stress-induced myocardial ischemia (MSIMI)^3–6^. Singular cardiovascular interventions often prove ineffective for MSIMI, necessitating concurrent psychological interventions to improve patient prognosis^7^. Therefore, accurate differential diagnosis between ANOCA and MSIMI is crucial for customized treatment and management strategies^8^.

Despite growing clinical awareness, the diagnosis of these conditions faces significant challenges, especially in resource-intensive primary healthcare settings. This is mainly due to the absence of specific, objective biomarker-based assessments to facilitate differential diagnosis. However, research into ANOCA and MSIMI is hampered by the lack of comprehensive datasets.

**Fig. 1:**
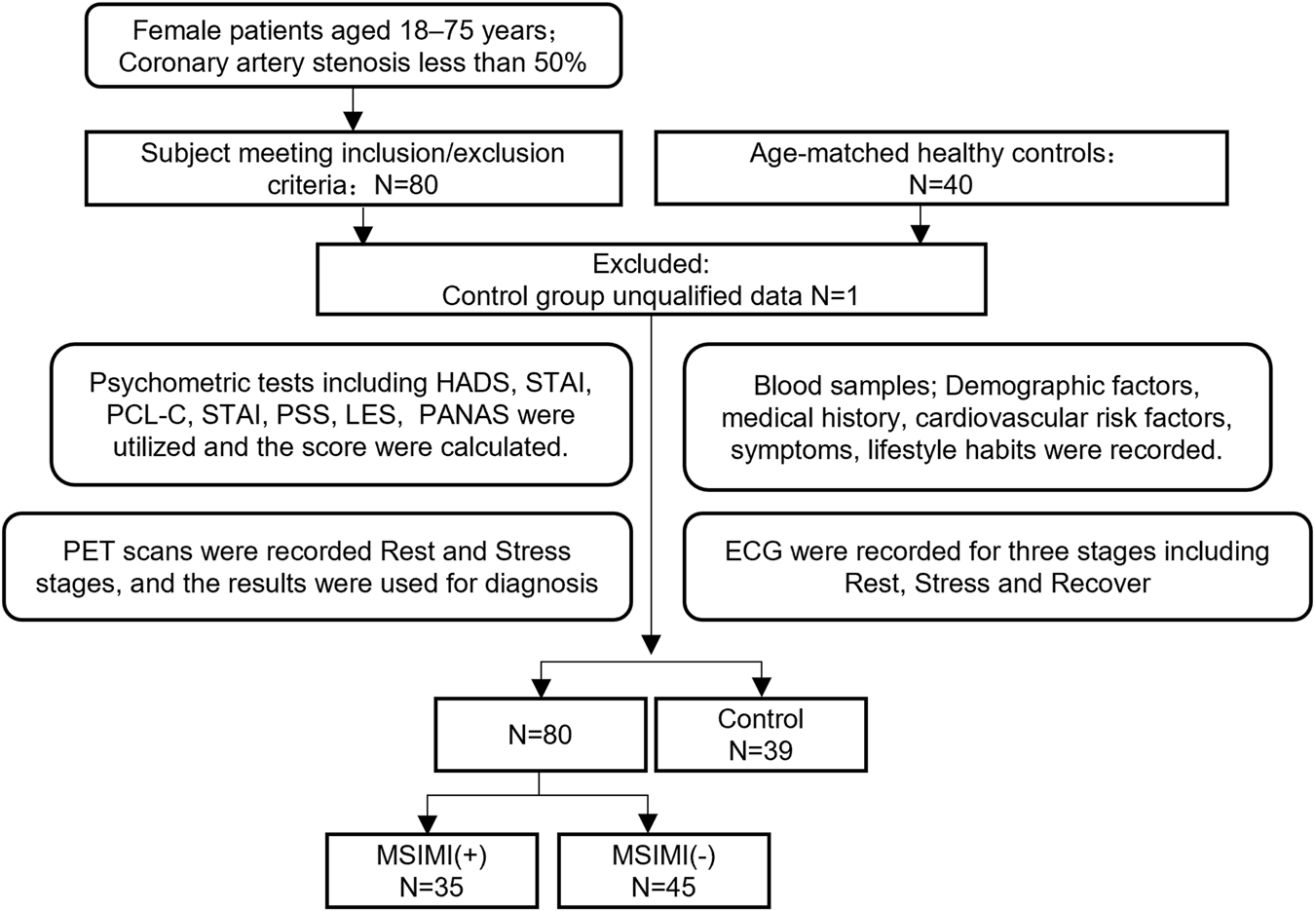
Flow of participants through the study.

**Fig. 2:**
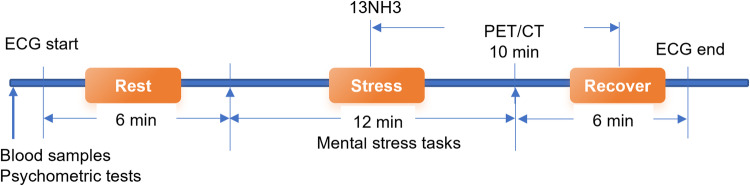
The process of data acquisition.

In this study, we prospectively recruited 80 female ANOCA patients and 40 age-matched healthy female controls. Each subject underwent three types of mental stress tasks in the laboratory (including mental arithmetic, public speech describing an event that occurred recently and was emotionally upsetting, and task-modified Stroop test) (Fig. 1). ECG data were continuously recorded throughout the entire experimental procedure, including the Rest, Stress, and Recover stages (Fig. 2) while PET/CT imaging, utilizing a ^13^N-ammonia tracer, was conducted seven minutes into the stress stage to assess myocardial perfusion. Diagnoses labels derived from ECG (by ST depression ≥0.1 mV) and PET/CT (by summed difference score, SDS ≥ 3) were made by experienced experts following established diagnostic guidelines^9^ (Fig. 1). PET/CT results were considered the gold standard for MSIMI diagnosis^10–13^. Physiological tests and blood samples were collected during the preparation period before mental stress tasks.

Given the clinical accessibility of ECG, we initiated our investigation with an analysis of ECG variables. A thorough review of the literature led to the selection of 88 explainable ECG variables^14–16^, encompassing well-established morphological variables and heart rate variability (HRV) metrics. HRV has been employed to examine the effects of mental stress on the autonomic nervous system^17–20^. The entirety of ECG data was divided into three sequential stages: Rest, Stress, and Recover, in accordance with the commencement and termination of mental stress tasks. Employing five conventional machine learning algorithms (Kneighbors, Logistic Regression, Random Forest, SVM, and XGBoost), we developed MSIMI diagnostic models based on ECG variables from various stages. The performances of these models suggested that ECG variables from different stages provided valuable insights for MSIMI classification, and stages such as Rest and Recover should not be disregarded in disease diagnosis. Our dataset further includes data from additional modalities, such as physiological tests and biochemical blood results. We anticipate that this multimodal dataset will significantly propel research forward in ANOCA and MSIMI, especially in addressing the complexities of differential diagnosis.

## Methods

### Participants

All experiments were conducted at Guangzhou’s Guangdong Provincial People’s Hospital in Guangzhou, Guangdong, China. The study was approved by the Ethics Committee at Guangdong Provincial People’s Hospital (approval ID: GDREC2019298H(R3)).

Participant selection adhered to established criteria:

Criteria for ANOCA inclusion:

- Women between the ages of 18 and 75 experiencing chest pain or similar symptoms of angina
- Coronary artery stenosis under 50% as confirmed by coronary CT angiography

Criteria for ANOCA exclusion:

- Chest pain attributable to non-cardiac circulatory system conditions
- New York Heart Association’s functional classification stage IV
- An obstructive myocardial infarction of the coronary artery in the preceding month
- An instance of apical ballooning syndrome in the preceding month
- Complications of serious illnesses, such as pulmonary embolism, severe arrhythmias, severe valvular heart disease
- Complications of severe psychiatric illness such as suicidal behaviors and cognitive impairments
- Current utilization of postmenopausal hormone treatment or psychotropic medication
- History of substance misuse, including alcohol or illegal drugs
- Engagement in other pharmacological studies in the last three months
- Current pregnancy or lactation

Concurrently, age-matched female control subjects, who do not experience chest pain and are free from coronary artery stenosis, will be sourced through diverse recruitment channels such as WeChat, public notices, online platforms, and radio broadcasts. To confirm the absence of obstructive CAD, control subjects will undergo a CT coronary angiography.

The hospital’s clinical care team identified and preliminarily discussed the study with prospective candidates. Interested individuals were then asked to sign informed consent forms.

### Procedures

Patients were hospitalised for the duration of the study. All laboratory tests were conducted between 7:00–10:00 AM while the participants were fasting. This was done to minimize the influence of circadian rhythms and ensure accurate comparison of ECG signals. Blood samples were collected during the preparation period. After a 6-minute supine rest, participants underwent three back-to-back mental stress tasks in a virtual reality setup within the diagnostic PET/CT unit. The tasks were: (1) a Stroop Color and Word Test; (2) a 3-minute speech on a distressing personal event to virtual doctors; and (3) rapidly performing serial subtractions of 7 from a three-digit number. Each task took 4 minutes, cumulating in a 12-minute comprehensive mental stress assessment. At the 7-minute mark of the stress stage, an injection containing 700–900 MBq of ^13^NH_3_ was used for myocardial perfusion evaluation and was administered in a “bolus-like” fashion (5 mL of ^13^NH_3_), immediately followed by a 10 mL NaCl flush (1 mCi corresponds to 37 MBq) to facilitate PET scanning. Beginning from a restful baseline, ECG monitoring was commenced and extended through six minutes post the mental stress examination, a process illustrated in Fig. 2.

### Data acquisition

#### ECG data

During the mental stress test, continuous ECG monitoring were conducted using the standard 12-lead ECG (Tim Software, Beijing Co., Beijing, China) with a 16-bit precision and a sampling frequency of 500 Hz.

#### PET/CT data

PET data acquisition and infusion of 13NH3 were commence after the CT scan for PET attenuation correction. All PET/CT examinations were conducted on a single clinical scanner (Biograph HI-REZ 16, Siemens Medical Solution) according to a standardized acquisition protocol, following international guidelines for PET/CT examinations.

#### Blood collection and tests

Blood samples were obtained on the day of the mental stress study before the mental stress test. The collected blood samples were analysed for cortisol, constituents of the renin-angiotensin-aldosterone system, adrenocorticotropic hormone, and thromboelastography. General and supplemental analyses were conducted to further examine our findings. These data could help to evaluate the pathogenesis of MSIMI from the perspectives of neuroendocrine mechanisms, sex hormone levels, humoral immunity index, and proteome expression.

#### Psychometric tests

Psychometric tests included the Positive and Negative Affect Schedule (PANAS), Hospital Anxiety and Depression Scale (HADS), Eysenck Personality Scale, Stress Perception Scale (PSS), State-Trait Anxiety Inventory (STAI), Life Event Scale (LES), Post-Traumatic Stress Disorder Checklist—Civilian Version (PCL-C).

### Data labeling and processing

#### ECG data

The diagnosis of MSIMI utilizing ECG criteria was established through consensus among three senior cardiologist, predicated on identifying ST depression exceeding 0.1 mV^11^. Each cardiologist had 10 or more years of clinical experience, and the final diagnosis was determined based on a consensus agreement among them. The MedEx MECG-200 ECG analysis system was used to filter the signal and characterize the heart’s electrical activity. Moreover, supplementary details were extracted from the hospital’s health record database, including patient’s unique ID, age and the date of data acquisition. The ECG recordings were divided into three parts, namely Rest, Stress, and Recover, based on the start and end times of the mental stress tasks as noted in the experimental records. The recordings were manually split and could be analysed separately or as a whole.

Noise caused by power line interference, baseline wander, and muscle contraction was removed using two median filters (200 ms, 600 ms) and the Daubechies wavelet with a decomposition tree of level 6. After the filtering process, wave peaks were detected, and morphological features and HRV indices were obtained using NeuroKit2, an open-source Python package suitable for both novice and advanced programmers. Information on how to install Python and NeuroKit2 can be found at https://github.com/neuropsychology/NeuroKit.

#### PET/CT data

PET data was acquired in list-mode and interpreted by two experienced readers following the recommendations of the American Society of Nuclear Cardiology. Prior to interpretation, PET images were screened for errors, such as patient movement, attenuation, reconstruction artifacts, and low count density. Perfusion were calculated using commercially available and previously validated software (QGS + QPS Automatic Quantification, Version 2013.1, Cedars-Sinai Medical Center, Los Angeles, USA). To quantify perfusion, a Summed Difference Score (SDS) was calculated as the difference between the summed stress score and the summed rest score. An SDS of 3 or greater is commonly accepted as indicative of MSIMI (+). Moreover, participants who experienced angina but recorded an SDS below 3 were categorized as MSIMI (−).

#### Other data

Blood test results, psychometric test results, and other relevant clinical data such as sleep quality and comorbidities were collected along with medical records and recorded in a Case Record Form (CRF) in compliance with Good Clinical Practice requirements. The CRF was used for future analysis.

## Data Records

The dataset is available for download from the Science Data Bank, as referenced in citation number^21^.

### Dataset overview

**Fig. 3:**
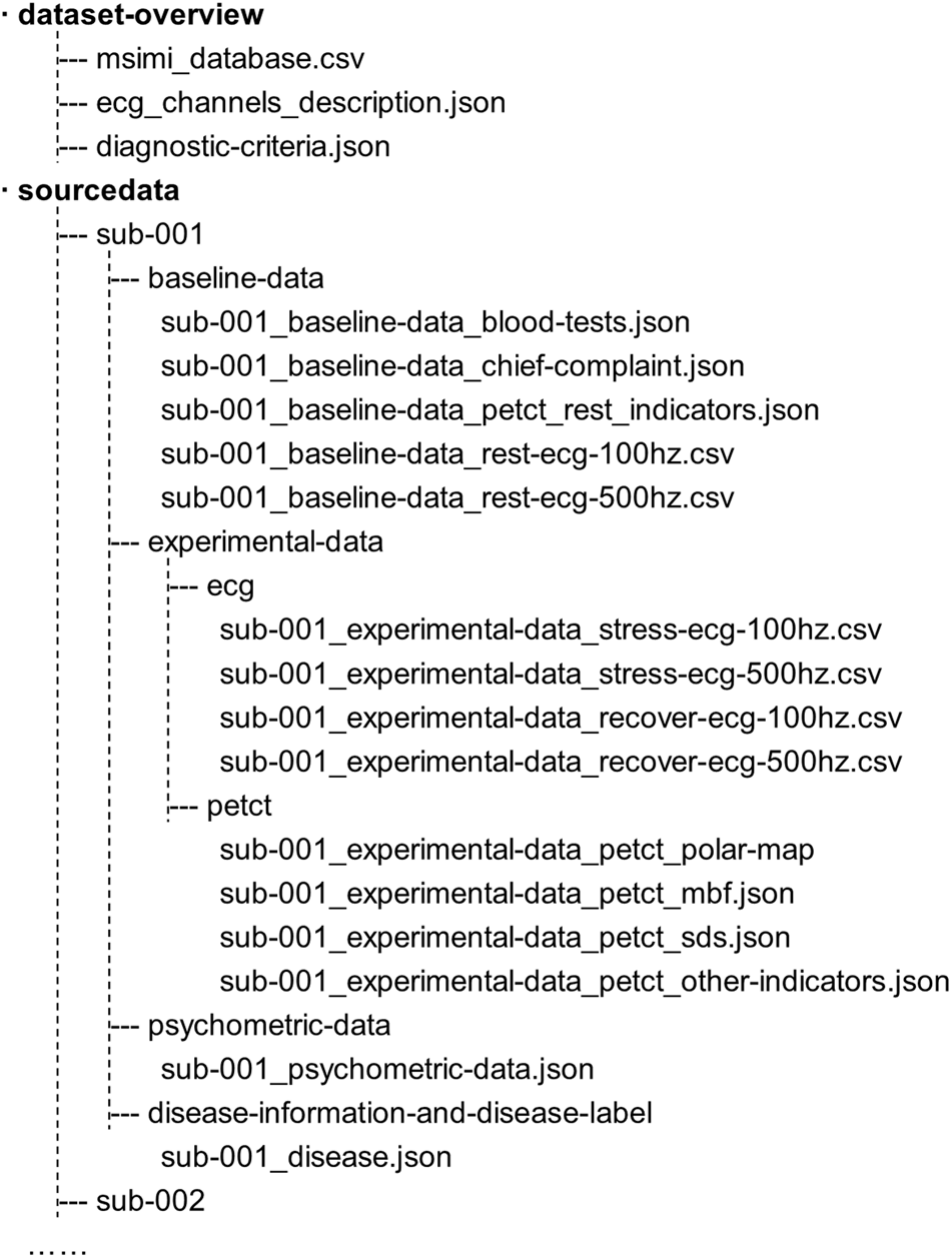
Directory tree for the repository with previews.

The dataset’s basic information, including dataset description, participant inclusion and exclusion criteria, disease definition, description of the mental stress task procedures, ECG channel descriptions, and diagnostic criteria based on PET/CT and ECG, were stored in separate files in the .json format^16^. Figure 3 shows the directory tree for our repository and previews of the meta-data.

### Dataset description

The ECG data for each participant during each session was saved in a ‘csv’ file format, with two different sampling frequencies available, namely 100 Hz and 500 Hz. The data for each subject (number: 001, 002,…) was stored as a first-level directory. The file naming rules were as follows.

Sub-xxx_baseline-data (or experimental-data or null) datatype (blood-tests, chief-complaint, petct_rest_indicators. *et al*.) where ‘xxx’ was the subject number (001, 002, …, 025).

The labels, including control, MSIMI (−), and MSIMI (+), are provided in sub-xxx disease.json. For specific diagnostic criteria, please refer to diagnostic-criteria.json (as seen in the PET/CT data section).

### Code availability

The code for dataset preparation is not intended to be released as it does not entail any potential for reusability.

### Source data

The source data was categorized according to the subject’s recorded stages, i.e., whether during the experiment or not. The baseline data consisted of blood test results, chief complaints, and ECG data recorded at rest before the mental stress tasks. The experimental data included ECG and PET/CT data recorded during the stress stages. The ECG data was the preprocessed raw data saved as ‘.dat’ files named after the subject number and stages. PET/CT data included polar maps, myocardial blood flow (mbf), and summed difference scores (SDS), the three key features for diagnosing MIMI. Among them, mbf is a vital prognosis indicator that has been established by numerous cardiology studies.

## Technical Validation

### Data quality assessment

To ensure the quality of the ECG recordings, we performed a signal quality assessment by computing established ECG morphology and heart rate variability (HRV) index from the original ECG recordings of the control group under resting conditions, as described in the Methods section. Subsequently, we compared these results with the standard criteria reported in previous studies. (Table 1)

**Table 1:** Comparation between calculation derived from the present dataset and standard criteria reported in previous studies.

Variable	Unit	Rest of the control (lead II)	medical reference range
P duration	s	0.084(0.017)	<0.11 s
PR interval	s	0.141(0.043)	0.12 ∼ 0.2 s
QRS duration	s	0.137(0.026)	Normal adult QRS time is less than 0.12 s, mostly between 0.06 ∼ 0.10 s.
QT interval	s	0.372(0.072)	0.32 ∼ 0.44 s
Pamp	mV	0.206(0.002)	Amplitude ≤ 0.25 mV in limb leads
Tamp	mV	0.210(0.004)	Amplitude < 0.5 mV in limb leads
SDNN	ms	102.8(62.5)	141 ± 39
pNN50	%	19.6(22.7)	16.7 ± 12.3
HTI		12.9(4.8)	≤20.42
LFHF		1.2(1.2)	Static supine position 5 min: 1.5 ∼ 2.0
LFnorm	nu	29(9)	Static supine position 5 min: 54 ± 24
HFnorm	nu	38(16)	Static supine position 5 min: 29 ± 3

### Disease classification

We conducted a detailed literature review to identify 88 ECG-based variables, including ECG morphology and heart rate variability (HRV) variables, which are listed in Table S1. The ECG recordings were extracted and divided into three specific stages according to the mental stress task’s timeline: Rest, Stress, and Recovery. During each stage, the ECG variables were independently calculated, resulting in a total of 264 variables (88 for each stage). Using variables from different stages and their combinations, we developed five classic machine learning models—K-Neighbors, Logistic Regression, Random Forest, SVM, and XGBoost—to distinguish MSIMI from control and ANOCA (diagnostic criteria see in PET/CT data section). The average accuracy of the models across different sets was obtained using leave-one-out cross-validation. The results indicate that ECG data from different stages can provide valuable information for MSIMI classification, and stages like Rest and Recover should not be overlooked during disease diagnosis.

## Usage Notes

This dataset has multiple potential uses for mental stress evaluation and daily ischemia detection. The hereby presented dataset and processing tools are provided for public use and may be used with proper citation to the current paper Table 2.

**Table 2:** Classification accuracy of algorithms using ECG data under different stages.

model	eval	Logistic Regression	SVM	Random Forest	Kneighbors	XGBoost
Complete	ROC	73.37	75.14	75.13	81.14	83.04
	acc	63.87	66.39	63.03	73.11	71.43
Rest	ROC	70.86	72.78	73.86	79.22	80.49
	acc	63.02	61.34	62.18	67.23	69.75
Stress	ROC	72.19	73.66	75.84	79.45	84.38
	acc	63.87	63.03	63.87	72.27	73.11
Recover	ROC	73.88	77.42	75.88	82.54	86.52
	acc	64.71	67.23	64.71	74.79	72.27
Rest + Stress + Recover	ROC	75.23	79.08	77.9	84.44	85.47
	acc	68.91	71.43	68.91	72.27	73.95

### Supplementary information

Table S1. ECG variables and its interpretation.

## Data Availability

For technical validation, we utilized publicly available code without any restrictions. Specifically, we employed the following functions/scripts: • **nk.ecg_peaks.py** from the NeuroKit2 package to identify R-peaks in an ECG signal (https://github.com/neuropsychology/NeuroKit). • **find_peaks.py** from the SciPy package to identify R-peaks in an ECG signal (https://docs.scipy.org/doc/scipy/reference/generated/scipy.signal.find_peaks.html). • **nk.ecg_delineate.py** from the NeuroKit2 package to delineate the QRS complex for morphology features (https://neuropsychology.github.io/NeuroKit/_modules/neurokit2/ecg/ecg_delineate.html#ecg_delineate). • **nk.hrv.py** from the NeuroKit2 package to compute Heart Rate Variability (https://neuropsychology.github.io/NeuroKit/_modules/neurokit2/hrv/hrv.html#hrv). • **linear_model.LogisticRegression**, **svm.SVC**, **ensemble.RandomForestClassifier**, **neighbors.KNeighborsClassifier**, and **XGBClassifier** from the Scikit-learn package for classification (https://scikit-learn.org/stable/). However, we customized and combined these packages to form our own code for the project. The Python program for ECG denoising and feature extraction is publicly available at https://github.com/pengxiaoting1995/MPPD_MSIMI.
