# Supplementary material for: A multimodal physiological and psychological dataset for human with mental stress induced myocardial ischemia
Source: Sci Data. 2024 Jun 27;11:704. doi: 10.1038/s41597-024-03462-2 (PMC11211436; doi:10.1038/s41597-024-03462-2)
Supplement: Supplementary file 1 — Table S1. ECG variables and its interpretation. [file 41597_2024_3462_MOESM1_ESM.docx]

Table S1. ECG variables and its interpretation.

| Type | Variable | Description | Interpretation |
| --- | --- | --- | --- |
| morphology | Pduration | P wave duration | An abnormally prolonged P wave duration may suggest impaired atrial conduction or enlargement of the atria, such as in cases of atrial fibrillation, atrial flutter, or other atrial arrhythmias. Additionally, an abnormally short P wave duration may indicate an accessory pathway or pre-excitation syndrome, such as in cases of Wolff-Parkinson-White (WPW) syndrome. |
| morphology | Tduration | T wave duration | a prolonged T wave suggest a prolonged QT interval, can be associated with conditions such as Long QT Syndrome, electrolyte imbalances, or certain medications |
| morphology | PRinterval | The PR interval is measured from the beginning of the P wave to the beginning of the QRS complex. | A prolonged PR interval duration may indicate first-degree AV block, which is often benign but can also be associated with underlying cardiac conditions such as myocarditis or Lyme disease |
| morphology | PRsegment | The PR segment is the flat, usually isoelectric segment between the end of the P wave and the start of the QRS complex. | May indicate certain cardiac conditions such as pericarditis or myocardial infarction. |
| morphology | QRSduration | QRS wave duration | A prolonged QRS duration may suggest bundle branch block, ventricular pacing, or certain cardiac conditions such as cardiomyopathy or myocardial infarction.  A shortened QRS duration may indicate Wolff-Parkinson-White (WPW) syndrome. |
| morphology | STinterval | The ST interval is the flat, isoelectric section of the ECG between the end of the S wave (the J point) and the beginning of the T wave. | ST segment elevation can be a sign of myocardial infarction, pericarditis, or early repolarization syndrome.  ST segment depression can be a sign of myocardial ischemia or hypokalemia. |
| morphology | STsegment | The ST segment connects the QRS complex and the T wave; it represents the period when the ventricles are depolarized. | ST segment elevation can be a sign of myocardial infarction, pericarditis, or early repolarization syndrome.  ST segment depression can be a sign of myocardial ischemia or hypokalemia. |
| morphology | TPinterval | The TP segment is the portion of the ECG from the end of the T wave to the beginning of the P wave. | Prolongation of the TP interval may be seen in conditions such as hypothermia, hyperkalemia, |
| morphology | QTinterval | The QT interval is measured from the beginning of the QRS complex to the end of the T wave. | Suggest the risk of developing torsades de pointes, a type of polymorphic ventricular tachycardia that can degenerate into ventricular fibrillation and cause sudden cardiac death |
| morphology | Pamp | The amplitude of P wave | Atrial enlargement, Atrial conduction abnormalities and other underlying heart disease |
| morphology | Qamp | The amplitude of Q wave | An indicator for various cardiac conditions such as myocardial infarction, cardiac hypertrophy, and bundle branch blocks. |
| morphology | Ramp | The amplitude of R wave | An indicator for cardiac hypertrophy, ventricular conduction abnormalities and myocardial infarction |
| morphology | Samp | The amplitude of S wave | A deep S wave can indicate right ventricular hypertrophy while a shallow S wave may indicate left ventricular hypertrophy or myocardial infarction. |
| morphology | Tamp | The amplitude of T wave | Low T wave can be seen in conditions such as hypokalemia (low potassium levels in the blood), myocardial ischemia, and myocardial infarction. |
| HRV | MeanNN | The mean of the NN intervals. | Has little to do with beat to beat variability and is simply the inverse of heart rate |
| HRV | SDNN | The standard deviation of the NN intervals. | The "gold standard" for medical stratification of cardiac risk when recorded over a 24 h period.  SDNN values predict both morbidity and mortality. |
| HRV | SDANN1 | The standard deviation of average NN intervals extracted from n-minute segments of time series data (1,2 and 5 by default). | same with the SDNN |
| HRV | SDNNI1 | Mean of the standard deviations of all NN intervals for all 5-minute segments of the entire recording (1,2 and 5 by default). | same with the SDNN  related to VLF power over a 24-h period |
| HRV | SDANN2 | The standard deviation of average NN intervals extracted from n-minute segments of time series data (1,2 and 5 by default). | same with the SDANN |
| HRV | SDNNI2 | Mean of the standard deviations of all NN intervals for all 5-minute segments of the entire recording (1,2 and 5 by default). | same with the SDNNI |
| HRV | SDANN5 | The standard deviation of average NN intervals extracted from n-minute segments of time series data (1,2 and 5 by default). | same with the SDANN |
| HRV | SDNNI5 | Mean of the standard deviations of all NN intervals for all 5-minute segments of the entire recording (1,2 and 5 by default). | same with the SDNN |
| HRV | RMSSD | The square root of the mean of the sum of successive differences between adjacent NN intervals. | Reflects the beat-to-beat variance in HR and is the primary time-domain measure used to estimate the vagally mediated changes reflected in HRV. The RMSSD typically provides a better assessment of RSA (especially in older subjects) |
| HRV | SDSD | The standard deviation of the successive differences between NN intervals. | Same with SDNN, only represents short-term variability |
| HRV | CVNN | The standard deviation of the NN intervals (SDNN) divided by the mean of the NN intervals (MeanNN) | Same with SDNN. |
| HRV | CVSD | The root mean square of the sum of successive differences (RMSSD) divided by the mean of the NN intervals (MeanNN). | Same as RMSSD. |
| HRV | MedianNN | The median of the absolute values of the successive differences between NN intervals. | Same as MeanNN |
| HRV | MadNN | The median absolute deviation of the NN intervals. | Same as MeanNN. |
| HRV | MCVNN | The median absolute deviation of the NN intervals (MadNN) divided by the median of the absolute differences of their successive differences (MedianNN). | Same as MeanNN. |
| HRV | IQRNN | The interquartile range (IQR) of the NN intervals. | Same as MeanNN. |
| HRV | pNN50 | The proportion of NN intervals greater than 50ms, out of the total number of NN intervals. | Closely correlated with PNS activity. It is correlated with the RMSSD and HF power. |
| HRV | pNN20 | The proportion of NN intervals greater than 20ms, out of the total number of NN intervals. | Same as pNN50 |
| HRV | HTI | The HRV triangular index, measuring the total number of NN intervals divided by the height of the NN intervals histogram. | HTI and RMSSD can jointly distinguish between normal heart rhythms and arrhythmias. When **HTI ≤ 20.42** and **RMSSD ≤ 0.068**, the heart rhythm is **normal**. When **HTI > 20.42, t**he pattern is arrhythmic |
| HRV | TINN | A geometrical parameter of the HRV, or more specifically, the baseline width of the NN intervals distribution obtained by triangular interpolation, where the error of least squares determines the triangle. It is an approximation of the NN interval distribution. | Same with SDNN, but not stable. |
| HRV | VLF | The spectral power of very low frequencies (by default, .0033 to .04 Hz). | Low VLF associated with #arrhythmic_death and PTSD, high inflammation, low levels of testosterone  The heart’s **intrinsic nervous system appears to contribute to the VLF rhythm** and the **SNS influences the amplitude and frequency of its oscillations**  PNS activity may contribute to VLF power since parasympathetic blockade almost completely abolishes it, sympathetic blockade does not affect VLF power |
| HRV | LF | The spectral power of low frequencies (by default, .04 to .15 Hz). | LF power may be produced by both the PNS and SNS, and BP regulation via baroreceptors, primarily by the PNS or by baroreflex activity alone.  **The LF band reflects baroreflex activity and not cardiac sympathetic innervation**. |
| HRV | HF | The spectral power of high frequencies (by default, .15 to .4 Hz). | The HF band reflects parasympathetic activity and is called the respiratory band because it corresponds to the HR variations related to the respiratory cycle.  **HF Power and RSA do not Represent vagal Tone** |
| HRV | VHF | The spectral power of very high frequencies (by default, .4 to .5 Hz). | The VHF presence in cardiac transplant patients and other conditions associated with reduced vagal influence on the heart. VHF activity of HRV is integral to the cardiovascular autonomic control. |
| HRV | LF/HF | The ratio obtained by dividing the low frequency power by the high frequency power. | A low LF/HF ratio reflects parasympathetic dominance. This is seen when we conserve energy and engage in tend-and-befriend behaviors. In contrast, a high LF/HF ratio indicates sympathetic dominance, which occurs when we engage in fight-or-flight behaviors or parasympathetic withdrawal. |
| HRV | LFn | The normalized low frequency, obtained by dividing the low frequency power by the total power. | Same with LF. |
| HRV | HFn | The normalized high frequency, obtained by dividing the low frequency power by the total power. | Same with HF. |
| HRV | LnHF | The log transformed HF. | Same with HF. |
| HRV | SD1 | Standard deviation perpendicular to the line of identity. It is an index of short-term RR interval fluctuations, i.e., beat-to-beat variability. | SD1 measures short-term HRV in ms and correlates with baroreflex sensitivity (BRS), which is the change in IBI duration per unit change in BP, and HF power. The RMSSD is identical to the nonlinear metric SD1, which reflects short-term HRV. SD1 predicts diastolic BP, HR Max − HR Min, RMSSD, pNN50, SDNN, and power in the LF and HF bands, and total power during 5 min recordings |
| HRV | SD2 | Standard deviation along the identity line. Index of long-term HRV changes. | SD2 measures short- and long-term HRV in ms and correlates with LF power and BRS. |
| HRV | SD1/SD2 | ratio of SD1 to SD2. Describes the ratio of short term to long term variations in HRV. | Measures the unpredictabilityof the RR time series, is used to measure autonomic balance when the monitoring period is sufficiently long and there is sympathetic activation. |
| HRV | S | Area of ellipse described by SD1 and SD2 (pi * SD1 * SD2). It is proportional to SD1SD2. | Correlates with baroreflex sensitivity (BRS) |
| HRV | CSI | The Cardiac Sympathetic Index is a measure of cardiac sympathetic function independent of vagal activity, calculated by dividing the longitudinal variability of the Poincaré plot (4*SD2) by its transverse variability (4*SD1). | CSI is an index of cardiac sympathetic function except in the resting supine condition, which is not affected by vagal activity. |
| HRV | CVI | The Cardiac Vagal Index is an index of cardiac parasympathetic function (vagal activity unaffected by sympathetic activity), and is equal equal to the logarithm of the product of longitudinal (4*SD2) and transverse variability (4*SD1). | CVI is a sensitive index of cardiac vagal function which is not affected by sympathetic activity |
| HRV | CSI_Modified | The modified CSI obtained by dividing the square of the longitudinal variability by its transverse variability. | Same with CSI |
| HRV | PIP | Percentage of inflection points of the RR intervals series. | The more fragmented a time series is, the higher the PIP indices will be. Heart rate fragmentation would be higher in healthy old subjects than in younger ones. |
| HRV | IALS | Inverse of the average length of the acceleration/deceleration segments. | The more fragmented a time series is, the higher the IALS indices will be. |
| HRV | PSS | Percentage of short segments. | The more fragmented a time series is, the higher the PSS indices will be. |
| HRV | PAS | Percentage of NN intervals in alternation segments. | The more fragmented a time series is, the higher the PAS indices will be. |
| HRV | GI | Guzik’s Index, defined as the distance of points above line of identity (LI) to LI divided by the distance of all points in Poincaré plot to LI except those that are located on LI. | ANS disorder, related to arrhythmias and heart failure. |
| HRV | SI | Slope Index, defined as the phase angle of points above LI divided by the phase angle of all points in Poincaré plot except those that are located on LI. | ANS disorder, related to arrhythmias and heart failure. |
| HRV | AI | Area Index, defined as the cumulative area of the sectors corresponding to the points that are located above LI divided by the cumulative area of sectors corresponding to all points in the Poincaré plot except those that are located on LI. | ANS disorder, related to arrhythmias and heart failure. |
| HRV | PI | Porta’s Index, defined as the number of points below LI divided by the total number of points in Poincaré plot except those that are located on LI. | ANS disorder, related to arrhythmias and heart failure. |
| HRV | C1d | The contributions of heart rate decelerations to short-term HRV | Significantly decreases in patients with chronic type 1 diabetes, heart failure and myocardial infarction. |
| HRV | C1a | The contributions of heart rate accelerations to short-term HRV. | Significantly decreases in patients with chronic type 1 diabetes, heart failure and myocardial infarction. |
| HRV | SD1d | Short-term variance of contributions of decelerations (prolongations of RR intervals). | Significantly decreases in patients with chronic type 1 diabetes, heart failure and myocardial infarction. |
| HRV | SD1a | Short-term variance of contributions of accelerations (shortenings of RR intervals. | Significantly decreases in patients with chronic type 1 diabetes, heart failure and myocardial infarction. |
| HRV | C2d | The contributions of heart rate decelerations to long-term HRV. | Significantly decreases in patients with chronic type 1 diabetes, heart failure and myocardial infarction. |
| HRV | C2a | The contributions of heart rate accelerations to long-term HRV. | Significantly decreases in patients with chronic type 1 diabetes, heart failure and myocardial infarction. |
| HRV | SD2d | Long-term variance of contributions of decelerations (prolongations of RR intervals). | Significantly decreases in patients with chronic type 1 diabetes, heart failure and myocardial infarction. |
| HRV | SD2a | Long-term variance of contributions accelerations (shortenings of RR intervals). | Significantly decreases in patients with chronic type 1 diabetes, heart failure and myocardial infarction. |
| HRV | Cd | The total contributions of heart rate decelerations to HRV. | Significantly decreases in patients with chronic type 1 diabetes, heart failure and myocardial infarction. |
| HRV | Ca | The total contributions of heart rate accelerations to HRV. | Significantly decreases in patients with chronic type 1 diabetes, heart failure and myocardial infarction. |
| HRV | SDNNd | Total variance of contributions of decelerations (prolongations of RR intervals). | Significantly decreases in patients with chronic type 1 diabetes, heart failure and myocardial infarction. |
| HRV | SDNNa | Total variance of contributions of and accelerations (shortenings of RR intervals). | Significantly decreases in patients with chronic type 1 diabetes, heart failure and myocardial infarction. |
| HRV | DFA_α1 | The monofractal detrended fluctuation analysis of the HR signal, corresponding to short-term correlations. | A strong predictor of both cardiac and total mortality. |
| HRV | DFA_α1_ExpRange | The monofractal detrended fluctuation analysis of the HR signal, corresponding to short-term correlations. | A strong predictor of both cardiac and total mortality. |
| HRV | DFA_α1_ExpMean | The monofractal detrended fluctuation analysis of the HR signal, corresponding to short-term correlations. | A strong predictor of both cardiac and total mortality. |
| HRV | DFA_α1_DimRange | The monofractal detrended fluctuation analysis of the HR signal, corresponding to short-term correlations. | A strong predictor of both cardiac and total mortality. |
| HRV | DFA_α1_DimMean | The monofractal detrended fluctuation analysis of the HR signal, corresponding to short-term correlations. | A strong predictor of both cardiac and total mortality. |
| HRV | DFA_α2 | The monofractal detrended fluctuation analysis of the HR signal, corresponding to long-term correlations. | A strong predictor of both cardiac and total mortality. |
| HRV | DFA_α2_ExpRange | The monofractal detrended fluctuation analysis of the HR signal, corresponding to long-term correlations. | A strong predictor of both cardiac and total mortality. |
| HRV | DFA_α2_ExpMean | The monofractal detrended fluctuation analysis of the HR signal, corresponding to long-term correlations. | A strong predictor of both cardiac and total mortality. |
| HRV | DFA_α2_DimRange | The monofractal detrended fluctuation analysis of the HR signal, corresponding to long-term correlations. | A strong predictor of both cardiac and total mortality. |
| HRV | DFA_α2_DimMean | The monofractal detrended fluctuation analysis of the HR signal, corresponding to long-term correlations. | A strong predictor of both cardiac and total mortality. |
| HRV | ApEn | Approximate entropy is a technique used to quantify the amount of regularity and the unpredictability of fluctuations over time-series data. | Lower in the major depressive disorder patients. |
| HRV | SampEn | Compute the sample entropy (SampEn) of a signal. SampEn is a modification of ApEn used for assessing complexity of physiological time series signals. | Lower in the major depressive disorder patients. |
| HRV | ShanEn | Compute Shannon entropy (SE/ShanEn). Entropy is a measure of unpredictability of the state, or equivalently, of its average information content. | Lower in the major depressive disorder patients. |
| HRV | FuzzyEn | Fuzzy entropy (FuzzyEn) of a signal stems from the combination between information theory and fuzzy set theory. A fuzzy set is a set containing elements with varying degrees of membership. | Lower in the major depressive disorder patients. |
| HRV | MSE | The multiscale entropy (MSEn), which computes sample entropies at multiple scales. | MSE complexity degrades with disease and aging. |
| HRV | CMSE | The Composite Multiscale Entropy algorithm, which computes multiple coarse-grained series for each scale factor (via the time-shift method for coarse-graining). | CMSE complexity degrades with disease and aging. |
| HRV | RCMSE | Refined CMSEn, which average not the entropy values of each subcoarsed vector, but its components at a lower level. | RCMSE complexity degrades with disease and aging. |
| HRV | CD | The Correlation Dimension (CD, also denoted D2) is a lower bound estimate of the fractal dimension of a signal. | Lower for patients who underwent acute and chronic mental stress. |
| HRV | HFD | The Higuchi’s Fractal Dimension (HFD) is an approximate value for the box-counting dimension for time series. It is computed by reconstructing k-max number of new data sets. | HFD values were found higher for diabetics than for healthy subjects. |
| HRV | KFD | Computes Katz’s Fractal Dimension (KFD). The euclidean distances between successive points in the signal are summed and averaged, and the maximum distance between the starting point and any other point in the sample. | KFD values were found higher for diabetics than for healthy subjects |
| HRV | LZC | Computes Lempel-Ziv Complexity (LZC) to quantify the regularity of the signal, by scanning symbolic sequences for new patterns, increasing the complexity count every time a new sequence is detected. | Lower values suggest unhealthy condition. |
